# Quality assurance for HIV point-of-care testing and treatment monitoring assays

**DOI:** 10.4102/ajlm.v5i2.557

**Published:** 2016-10-17

**Authors:** Adrienne F.A. Meyers, Paul Sandstrom, Thomas N. Denny, Mackenzie Hurlston, Terry B. Ball, Rosanna W. Peeling, Debrah I. Boeras

**Affiliations:** 1 QASI, National HIV & Retrovirology Laboratories, Public Health Agency of Canada, JC Wilt Infectious Diseases Research Centre, Winnipeg, Manitoba, Canada; 2 Department of Medical Microbiology and Infectious Diseases, University of Manitoba, Winnipeg, Manitoba, Canada; 3 Department of Medical Microbiology, University of Nairobi, Nairobi, Kenya; 4 Duke University, Department of Medicine, Durham, North Carolina, United States; 5 Centers for Disease Control and Prevention, Atlanta, Georgia, United States; 6 Department of Immunology, University of Manitoba, Winnipeg, Manitoba, Canada; 7 London School of Hygiene & Tropical Medicine, London, United Kingdom

## Abstract

In 2015, UNAIDS launched the 90-90-90 targets aimed at increasing the number of people infected with HIV to become aware of their status, access antiretroviral therapies and ultimately be virally suppressed. To achieve these goals, countries may need to scale up point-of-care (POC) testing in addition to strengthening central laboratory services. While decentralising testing increases patient access to diagnostics, it presents many challenges with regard to training and assuring the quality of tests and testing. To ensure synergies, the London School of Hygiene & Tropical Medicine held a series of consultations with countries with an interest in quality assurance and their implementing partners, and agreed on an external quality assessment (EQA) programme to ensure reliable results so that the results lead to the best possible care for HIV patients. As a result of the consultations, EQA International was established, bringing together EQA providers and implementers to develop a strategic plan for countries to establish national POC EQA programmes and to estimate the cost of setting up and maintaining the programme. With the dramatic increase in the number of proficiency testing panels required for thousands of POC testing sites across Africa, it is important to facilitate technology transfer from global EQA providers to a network of regional EQA centres in Africa for regional proficiency testing panel production. EQA International will continue to identify robust and cost-effective EQA technologies for quality POC testing, integrating novel technologies to support sustainable country-owned EQA programmes in Africa.

## The need for quality assurance

It is estimated that 36.9 million people globally are living with HIV, with more than two-thirds of these individuals located in resource-limited regions.^1^ The ambitious UNAIDS 90-90-90 targets, aimed to end the HIV epidemic within the next 15 years, place diagnostics in a key role for achieving these goals: by 2020, 90% of all people living with HIV will know their HIV status; 90% of all HIV-positive people will receive sustained antiretroviral therapy; 90% of all people receiving antiretroviral therapy will achieve viral suppression.^2^ Recent estimates suggest that 15.8 million people are receiving antiretroviral therapy. With the new World Health Organization guidelines recommending test and treat, it is expected that 30 million people living with HIV will have access to treatment by 2020.^3^

The achievement of the 90-90-90 targets depends on the availability of tests for HIV case detection, including early infant diagnosis (EID) and viral load for monitoring viral suppression. CD4 enumeration is still used for treatment initiation in many countries as they move toward the World Health Organization guidelines of test and treat, and plan to scale up viral load testing. However, access to these tests is reduced in resource-limited settings. Recent efforts aimed at improving access to these assays have seen a large increase in novel technologies that can be performed at the point of care (POC). The rollout of rapid POC tests for HIV testing and treatment initiation has significantly reduced the time for patients to receive results and initiate therapy.^4,5^ As testing is decentralised to hundreds of sites outside of the laboratory, and being performed by non-laboratory staff, ensuring test quality and accuracy of results have become key considerations in HIV programmes.^6^

## Current point-of-care technologies for CD4, early infant diagnosis and viral load testing

To date, there are two approved (World Health Organization-prequalified) POC platforms for CD4 and two for EID, whereas POC viral load platforms are in the evaluation pipeline.^7^ Most of these POC technologies provide sample-in/answer-out diagnostic testing, making it possible for these tests to be performed with minimal training, outside of the traditional laboratory setting. Most of the devices can withstand extreme transport and environmental conditions, provide a test result in less than one hour and have data transmission capabilities. Some (and more are in development) can even operate on battery power.^8^

The rollout of CD4 POC devices in the last few years has provided us with lessons learnt in the implementation and quality management for POC testing. EID and viral load POC testing has not yet been fully implemented in the field, but will benefit from lessons learned from the CD4 experience to strengthen existing quality programmes and ensure integration of POC testing within these programmes.

## Understanding quality assurance

**FIGURE 1:**
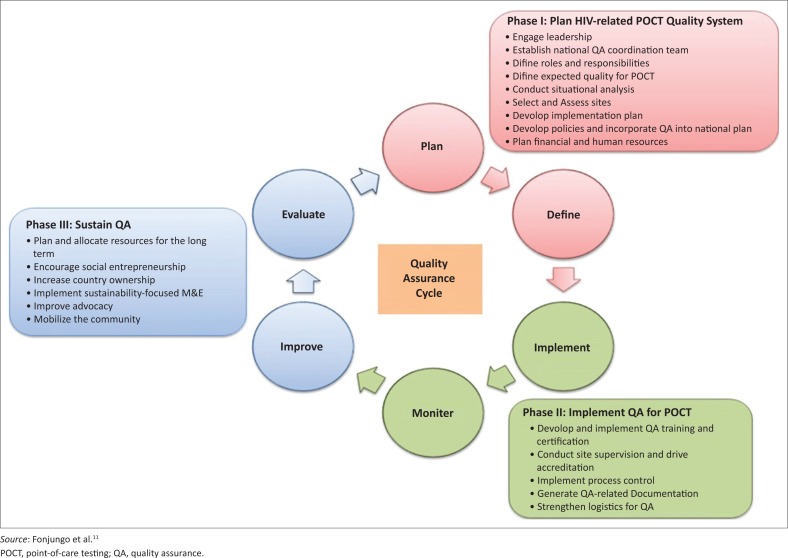
World Health Organization/US Centers for Disease Control and Prevention quality assurance cycle to improve the quality of HIV-related point-of-care testing.

Quality management is the entire process supporting the quality of testing, while quality assurance is the planned activities implemented to support the quality management system (Figure 1).^9,10^ While it is highly recommended that a POC quality assurance plan address the entire quality assurance management cycle, in this report we focus on external quality assurance as a quality assurance activity relevant to POC testing, and the results serve as a strong indicator of laboratory quality.^11^

POC devices have in-built internal quality controls to monitor in real time the performance of an instrument and, to some extent, the user. If any of the controls fail, the device should not provide a test result. In contrast, external quality assessment (EQA), often used interchangeably with proficiency testing, challenges the testing environment and allows for an external expert body to examine the processes and provide remedial action.^9,12,13^

The three components of an effective EQA laboratory programme are site supervision, retesting of specimens, and proficiency testing.^14^ Supervision is integral to quality assurance and should be done routinely, but with minimal disruption to patient services. Retesting is rarely done due to costs and time constraints, and is especially challenging in the POC environment. Proficiency testing is the most widely-used approach to monitor the performance of the test used and the quality of testing. It involves blinded control material, meant to mimic patient specimens, sent from a reference laboratory to the testing site and results sent back for scoring. Participation in EQA programmes is often mandated for clinical laboratories performing regular testing of patient samples for HIV care and management.^15,16^

A low proficiency testing score should alert the laboratory or testing site that there is a problem. Often, identifying the exact nature of the problem, and then correcting the problem, is difficult, even for skilled technicians. The error can be either an instrument error or an operator error. Investigation and remedial action should be taken immediately to correct the problem before patients’ results are adversely affected.

## Current external quality assessment schemes

A comprehensive external quality assurance programme consists of the following: proficiency testing panel design and production, EQA scheme/survey, results and reporting, customer service/technical support, and corrective action (Table 1).

**TABLE 1:** Considerations for external quality assessment programmes.

EQA Activity	Considerations for EQA Programme
Proficiency Testing materials/specimens	Compatible with the testing technology. Ideally mimic the actual patient specimen as closely as possible. Well-characterised and stable in order to reach the testing facility. Homogenous to ensure participants in the EQA programme receive similar testing material. Should not be cost-prohibitive.
EQA survey	Multiple blinded samples provided periodically to a sufficient group of participating laboratories for robust analysis and/or identification (CLSI GP27-A2 27:8).^12^ Proficiency testing should be provided in a frequency that measures the laboratory capability. Minimum frequency should be twice per year,^15,26^ while 3–4 times per year may be optimal. CD4 providers range in surveying from 2–6 times per year with size of panels ranging just as much. EID and viral load EQA are typically provided in 2–6 panels per year.
Results and reporting	Timely reporting of results, possibly using reporting mechanisms that mimic patient reporting. Clear concise reports, should include: results of reporting participant method of measurement (i.e. instrument used) target values expected for each analyte measured results distribution for all participants in the session, and acceptability of reported results (satisfactory/unsatisfactory) pass/fail. Final EQA report compares the performance of the participant to others participating in the same programme.
Customer service/Technical support	Important to the overall process and leads to improvement of participating sites. EQA provider should provide reliable services to their customers, e.g. providing alternative innovative means for communication to participants in remote sites with unreliable internet access (SMS, WhatsApp). Annual letters with calendar of EQA surveys and updates to the programme.
Corrective action	Allows for the identification and correction of matters related to a variety of quality issues ranging from staff and instrument performance to infrastructure challenges, such as stock outs and transport issues. Opportunity for refresher trainings. Improved performance.

EQA, external quality assessment; EID, early infant diagnosis.

An EQA survey or cycle consists of a panel of specimens intended to challenge or assess the testing event. EQA should, as far as possible, cover the entire range of tests, and the entire examination process, from sample reception, preparation and analysis to interpretation and reporting.^17^ The goal is to allow for regularity in testing that will highlight any changes or challenges a participant may encounter without having a long period of inaccurate testing going undetected. The reporting of results, as well as immediate corrective action to improve programmes, is the most critical component to any EQA programme. Reporting results is a major challenge in resource-limited settings due to various reasons that result in wasted resources and systematic problems going undetected. The number of specimens or members in each panel and frequency of EQA often varies by type of test and the EQA provider. Programmes often struggle to find an overall balance between ‘necessary’ and ‘sufficient’ in order to minimise costs and maximise resources.

## External quality assessment for point-of-care CD4, early infant diagnosis and viral load testing

As new technologies are introduced, EQA needs to evolve to accommodate new testing strategies.^18^ In an effort to address the topic of quality of testing at hundreds, perhaps thousands of sites in a country, this section will consider what is necessary to ensure quality POC CD4, EID, and viral load testing.

### Projected demands for point-of-care testing

With ‘test and treat’ and the scale up of viral load testing for monitoring patients, CD4 volumes are projected to remain the same in the short term and eventually decline as programmes transition to viral load testing. While POC testing for EID and viral load is currently not offered in some countries, planning for the rollout of these tests is ongoing and presents a critical time to develop strategies for quality assurance of these tests. To ensure synergies, the London School of Hygiene & Tropical Medicine established EQA International, bringing together EQA providers and implementers to develop a strategic plan for working with countries in Africa to establish national POC EQA programmes and estimate the cost of setting up and maintaining the programme. Members of EQA International include The Public Health Agency of Canada QASI programme, the US Centers for Disease Control and Prevention, Duke University External Quality Assurance Program Oversight Laboratory and Immunology Quality Assessment, South Africa National Health Laboratory Service, Zimbabwe National Quality Assurance Programme and Foundation Mérieux. EQA International is working closely with funders and implementing partners to remain one step ahead of the phased rollout of POC testing for EID and viral load in Africa.

### How to deliver on the demands of quality-assured point-of-care testing

Assuming hundreds and possibly thousands of devices will be placed in Africa over the next five years, EQA International, in consultation with national HIV programme and laboratory managers from countries in Africa with an interest in participating in EQA programmes for POC testing, agreed on a work plan for the standardisation, production and distribution of proficiency testing panels and reporting mechanisms, to cover CD4, EID and viral load POC testing in Africa (Table 2).

**TABLE 2:** Proposed external quality assessment scheme for CD4, early infant diagnosis and viral load point-of-care testing.

Test	EQA Survey	EQA Panel
CD4	3× per year	2-member panel: high and low concentration
EID	2× per year	3-member panel: 1 negative and 2 positive samples
Viral load	2× per year	3-member panel: 1 negative, 2 positives (1 high and 1 low)

Note: Shipments will be combined to save costs.

EQA, external quality assessment; EID, early infant diagnosis.

## External quality assessment technology

Ideally, EQA proficiency testing specimens can be used for both POC and laboratory testing. While the use of fresh whole blood samples most closely resembles clinical samples tested by a participant, if an EQA programme has several hundred participants or a participant base located over a wide geographical area, it may not be feasible to access enough material and distribute to participants within the time-frame that the material is stable and effective for proper evaluation. Commercially-stabilised whole blood products, while financially more challenging, are homogeneous and stable for weeks to months, which is beneficial for large EQA programmes.

Novel technologies are likely to be needed, given the volumes and potential logistical challenges, such as maintenance of sample integrity during transport. Several innovative cost-effective applications are being explored to provide stabilised EQA specimens for CD4, EID and viral load testing. A major challenge with CD4 has been the necessity for whole blood and therefore stabilisers are required as a means of extending pre-analytic capacity for CD4 T-cell enumeration.^19^ Considering POC sites, an ideal candidate would allow the EQA CD4 and EID specimens to remain stable for up to three weeks at ambient temperatures in-country.

Another innovative technology, the dried tube specimen (DTS) has been successfully used for EQA for HIV and syphilis rapid diagnostic tests and for automated viral load laboratory tests.^20,21,22,23,24^ DTS can likely be expanded for EQA of viral load POC tests as well as other tests. A study specifically looking at the relative impact of costs of implementing DTS EQA with the rollout of a rapid syphilis testing programme found the incremental costs of EQA relatively small and further cost reductions were possible through programme redesign.^25^

EQA sample considerations for POC CD4, viral load and EID programmes are summarised in Table 3. EQA International is working to identify robust and cost-effective EQA technologies for quality POC testing and integrating novel technologies to support sustainable EQA programmes.

**TABLE 3:** External quality assessment sample considerations.

Sample indicators	Proficiency testing source materials
Appropriate matrix	Should mimic patient specimen; use locally sourced fresh whole blood; should be available in large volumes; dried tube specimens can be used as proficiency testing for viral load.
Stable, homogeneous material	Stabilising agent should not modify the diagnostic target molecules; should allow the panel to be stable for 3 weeks at ambient temperature.
Appropriate concentration levels	Concentrations should include clinically-relevant levels.
Appropriate frequency of testing	Consider starting with 2 surveys per year and increase coverage in country before increasing frequency. Recommendations by the American Association for Laboratory suggest that laboratories should be compared at least twice annually to the laboratory comparator for continued performance evaluation.^26^

## Regional external quality assessment coordination centres

Currently, the majority of proficiency testing panels are procured from commercial EQA providers outside Africa. Shipment of proficiency testing panels to individual laboratories is costly and logistical challenges are frequently encountered.

A regional approach would allow for distribution of a well-characterised panel of sufficient sample size to be used to compare performances across the region. The National Health Laboratory Service in South Africa is currently providing EQA for EID to more than 10 neighbouring countries. The AFRIQUALAB in Senegal supports EQA for CD4 and EID to more than 40 countries. The Zimbabwe National Quality Assurance Program is establishing a regional EQA centre to support CD4, EID and viral load testing. Finally, the African Society for Laboratory Medicine has recently established a network of six collaborating centres across Africa to strengthen laboratory medicine; these collaborating centres could also function as regional EQA centres.

**FIGURE 2:**
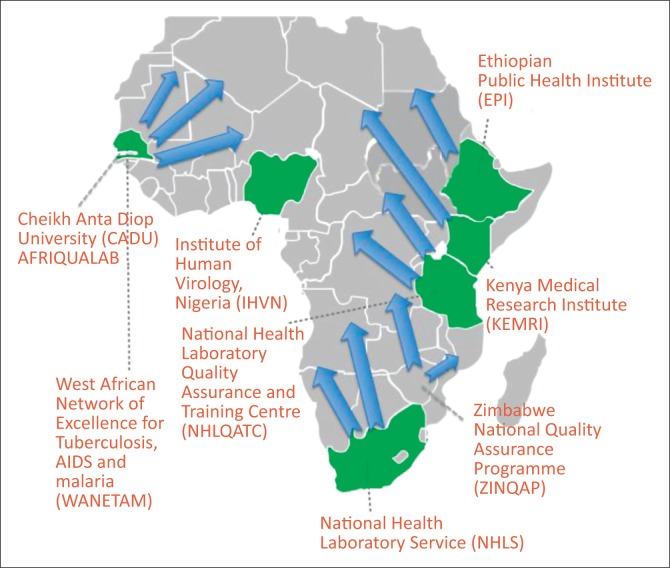
Global external quality assessment coverage through regional external quality assessment centres.

A network of regional EQA coordinating centres can be set up to produce or procure proficiency testing materials from global providers, and coordinate distributions (Figure 2). These centres could also oversee the logistics and management of the EQA programme, providing timely corrective action and strengthening national quality assurance programmes and country capacity. Through technology transfer, these centres could produce panels at minimal cost, using locally-sourced material.

Regional EQA coordinating centres can also support other programmes and services. The World Health Organization Regional Office for Africa has linked EQA programmes for their laboratory networks for influenza, malaria, meningitis, enterics and other diseases.^26^ The East African Regional External Quality Assessment Scheme supports EQA in five countries to improve the quality of essential diagnostic services in clinical laboratories at peripheral level. Future capacities of regional EQA centres could serve as biobanking facilities that support test performance evaluations and validations, post-market surveillance, global health surveillance, and facilitate new test development.

## Benefits of external quality assessment programmes

There is strong evidence to indicate that participation in EQA programmes significantly improves testing performance.^23,26,27^ The US Centers for Disease Control and Prevention has developed EQA programmes to help participating laboratories monitor the quality of laboratory-based viral load and EID testing in low- and middle-income countries. In an analysis of the reported EQA data from more than 114 participating laboratories in 44 countries in the viral load programme, and 136 participating laboratories in 41 countries in the EID programme, they found that with increased participation came increased performance.^23,27^ The Public Health Agency Canada (QASI)-Quality Management System, designed to work globally, optimised its programme strategies in 2009 and immediately saw reduced error rates with their CD4 programme as well as significant reduction in inter-laboratory variability resulting from continuous participation in the QASI-quality management system.^28^ QASI provides detailed, extensive technology transfer workshops to programme participants using a ‘train-the-trainer’ format, which significantly improves participation and performance. With CD4, improvements in performance of programmes can be directly linked to improvements in patient care.^4,11^

Apart from insights into the participants’ performance levels, EQA can also survey the overall national laboratory infrastructure and trace problems in processes. Examples of this include the sample transport network and supply chain management system. These two systems often go hand-in-hand and contribute to reagents/kit stock-outs and poor quality of specimens. A comprehensive, coordinated EQA programme can identify these gaps and bring them to the attention of the country’s Ministry of Health and partners for immediate corrective action, strengthening the overall infrastructure of the country and thereby improving programmes and services. In addition, EQA programmes can also capture key indicators and document programme improvements over time.

EQA is an important tool for providing the educational stimulus that will improve overall performance and standards.^29^ Other quality assurance activities, such as training and site evaluations, can support a quality-assured environment.^10,30^ EQA also builds educational stimulus and promotes research capacity through technology transfer and trainings. The Institute of Public Health in Brussels, Belgium looked at EQA for malaria rapid diagnostic tests and found that EQA prompted educational incentives that boosted self-confidence and credibility.^31^ EQA can also provide valuable information to programmes and manufacturers though post-market surveillance.^32^ Post-market surveillance ensures that diagnostics continue to meet quality, safety and performance requirements. Often failures go unreported, but though a proactive EQA programme, these failures can be captured and reported appropriately.

**FIGURE 3:**
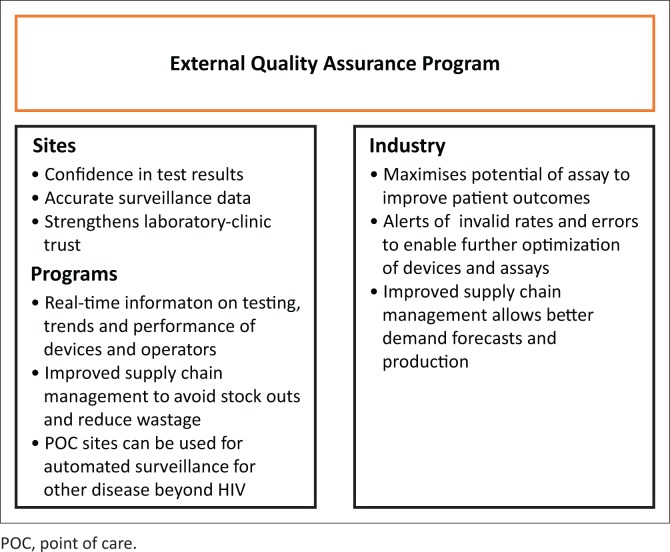
Benefits of an external quality assurance programme to point-of-care sites, country programmes, and point-of-care industry.

Participation in an external quality assessment program provides many benefits and valuable data to health authorities (Figure 3):^30,33^

- Comparison of performance and results among different test sites.
- Collection of data across different sites.
- Early warning for systematic problems.
- Objective evidence of testing quality.
- Indicates areas that need improvement.
- Survey the use of diagnostic algorithms.
- Potential to build health security through surveillance and monitoring.

A key feature of many of these POC molecular devices is that they are equipped with data transmission capacities. Apart from monitoring the quality of tests and testing to optimise supply chain management, data connectivity links data from diagnostic laboratories and POC test readers and devices to provide data on testing coverage, disease trends and timely information for early warning of infectious disease outbreaks.^34^

## Looking forward: Sustainable, country-owned external quality assessment programmes in Africa

Sustainability of EQA programmes requires country ownership. As countries in Africa plan the scale-up of POC technologies for CD4, viral load and EID testing, political commitment toward quality should be reflected in country policies and implementation plans. Putting in place EQA programmes that can identify gaps and challenges in the testing environment and address them immediately to increase the efficiency of healthcare systems and improve patient results, are urgent priorities.

The role of implementing partners and industry in support of EQA and quality assurance programmes is also a strong consideration for the sustainability of quality-assured testing. Partners and industry can bring numerous resources to these programmes. As an example, when Zimbabwe was establishing their national quality assurance programme, the Ministry of Health brought together stakeholders as well as quality assurance and POC partners to share their vision for a coordinated national programme that would identify redundancies and gaps and leverage partner resources and commitment. This process secures a sustainable country-owned quality assurance national policy.
